# The efficacy of velar adhesion in unilateral cleft lip and palate patients: Cleft width and otitis media with effusion

**DOI:** 10.1371/journal.pone.0323503

**Published:** 2025-05-28

**Authors:** Miki Kashiwagi, Hideto Saijo, Sachi Oshima, Shota Ichikawa, Rika Narita, Kazuto Hoshi

**Affiliations:** 1 Department of Oral and Maxillo-Facial Surgery and Orthodontics, The University of Tokyo Hospital, Tokyo, Japan; 2 Department of Oral and Maxilloacial Surgery, The University of Osaka, Osaka, Japan; 3 Department of Oral and Maxillofacial Surgery, Field of Oral and Maxillofacial Rehabilitation, Advanced Therapeutics Course, Graduate School of Medical and Dental Sciences, Kagoshima University, Kagoshima, Japan; 4 Center for Cleft Lip and Palate, The University of Tokyo Hospital, Tokyo, Japan; University of the Republic Uruguay: Universidad de la Republica Uruguay, URUGUAY

## Abstract

**Background:**

Cleft lip and palate (CLP) is one of the most common congenital anomalies, affecting ~1 in 700 births worldwide. Patients with CLP often experience functional impairments due to the cleft palate, particularly related to feeding and speech. Surgical interventions are essential for addressing these issues, yet no standardized surgical procedure exists. Velar adhesion (VA) is a technique used to reduce the cleft width prior to a palatoplasty, potentially improving surgical outcomes, but its effectiveness remains unclear.

**Objective:**

The impact of VA on cleft-width reduction and the incidence of otitis media with effusion (OME) were evaluated in patients with unilateral cleft lip and palate (UCLP).

**Patients and methods:**

The cases of 45 patients with UCLP who underwent a palatoplasty at the University of Tokyo Hospital between January 2013 and December 2023 were analyzed retrospectively. We divided the patients into two groups: those who underwent VA during lip repair (VA group) and those who did not (non-VA group). The cleft width and alveolar cleft width were measured at birth, lip repair, and palatoplasty. The presence of OME was assessed the day before the palatoplasty. Pearson’s chi-square test and the two-tailed t-test were applied.

**Results:**

Significant cleft-width reduction was observed in the VA group compared to the non-VA group at the time of palatoplasty (4.58 mm vs. 6.55 mm, p < 0.01). The incidence of OME was significantly lower in the VA group (60.00%) versus the non-VA group (90.91%, p < 0.01). No significant between-group differences were identified for the alveolar cleft width or maxillary growth.

**Conclusion:**

VA significantly reduces the cleft width at the junction of the hard and soft palates, and it may decrease the incidence of otitis media with effusion in patients with UCLP. VA is a straightforward procedure with potential benefits for improving palatoplasty outcomes and mitigating complications such as OME.

## Introduction

Cleft lip and palate (CLP) is the most common congenital anomaly among external bodily deformities. Worldwide, CLP occurs at a rate of 1 in 700 births [1]. The clinical presentation of individuals with CLP varies, and cleft palate in particular can cause functional impairments related to feeding and speech [2–4]. The impairments that are associated with cleft palate include abnormalities in maxillary development [5], ear disorders such as otitis media with effusion (OME), and psychological issues [6–8]. It has also been reported that in individuals with CLP, OME can lead to long-term hearing and speech impairments [9].

Several surgical interventions and techniques for correcting a cleft palate have been extensively reported, but there is no standardized or universally accepted procedure. The reported techniques include the von Langenbeck method [10], the two-flap method [11], the Wardill-Kilner Pushback method [12], the Furlow double opposing Z-plasty [13,14], and a two-stage palatoplasty [15], each with its own advantages and disadvantages. Regardless of the surgical technique used, it is crucial to avoid postoperative fistula formation and ensure the acquisition of reliable velopharyngeal closure function. It is thus desirable for surgeons to operate on an optimal maxillary morphology, which facilitates the success of the palatoplasty.

Perhaps because narrower cleft widths entail less tissue tension, performing a palatoplasty is generally considered to be easier when dealing with narrower cleft widths. One method to narrow the cleft width prior to a palatoplasty is through velar adhesion (VA) [16], which is a surgical procedure that involves suturing the soft palate during cleft lip repair, prior to the palatoplasty. By performing VA, the width of the cleft palate is reduced before the palatoplasty, easing the mucosal tension and facilitating the subsequent palate formation surgery [16]. However, only a few reports concerning the use of VA are available, and most of them have very small sample sizes; e.g., about a dozen cases. The details regarding the effectiveness of VA have thus been unclear.

Individuals with CLP have a higher likelihood of developing OME due to the anatomical structure involved [2]. The reason for the increased incidence of OME in people with a cleft palate has been thought to be the exposure and potential contamination of the eustachian tube orifice, but during the application of VA, there are alterations in the anatomical structure near the eustachian tube orifice, suggesting that the use of VA may influence the incidence of OME. We conducted the present study to determine whether the occurrence of OME after cleft lip repair is affected by the use of VA.

## Patients and methods

### Sample characteristics

This study was a retrospective analysis of the medical records and dental models of patients with unilateral cleft lip and palate (UCLP). The study population was 67 patients who underwent a palatoplasty at the Department of Oral and Maxillofacial Surgery and Orthodontics, University of Tokyo Hospital during the period from January 2013 to December 2023. The University of Tokyo Hospital ethical committee approved this study (Ethics approval no. 2945-16). All procedures performed herein followed the ethical standards of University of Tokyo Hospital.

We accessed the data for research purposes from October 1, 2023, to January 31, 2024. We analyzed the clinical data in an anonymized state, and access to the anonymized data was possible both during and after the data collection. We excluded the cases of patients whose dental cast models were not available and those in which the velar adhesion was lost postoperatively. After the exclusion of those cases, 41 cases remained as the subjects of investigation. The patients were 27 males and 14 females, all diagnosed with UCLP.

### Surgical methods

**Fig 1:**
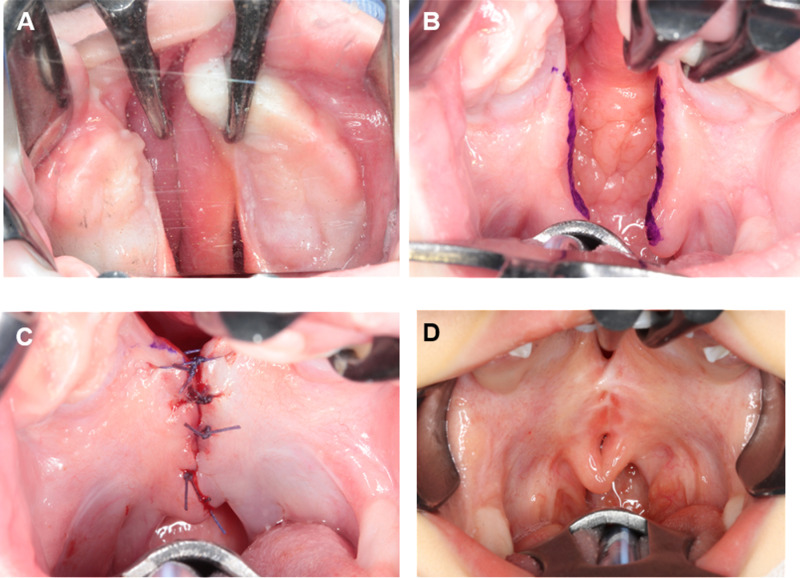
Velar adhesion (VA). (A) Incision line of hard palate, (B) incision line of soft palate, (C) sutured by absorbable thread, and (D) adhesion of soft palate at the time of palatoplasty.

At our hospital, the use of a Hotz plate is required for infants with CLP, as soon as possible after birth. The plate is used until the cheiloplasty at 3–6 months of age. In most of the present study’s cases, the palatoplasty was performed around the age of 15–18 months. Velar adhesion was performed simultaneously with the patient’s cheiloplasty. The cleft margin of soft palate mucosa was incised around 4–5 mm from the cleft margin. The incision of the cleft margin was made from the level of the maxillary tuberosities to the base of the uvula (Fig 1A–D). Three or four sutures with absorbable threads were applied on the nasal and oral side, respectively. At this point in the surgery, special care was taken to avoid excessive mucosal tension. In the cases in which VA was applied, the cheiloplasty was performed after the VA was completed. After VA and until the palatoplasty, the patients were not required to use any plates including the Hotz palate. Approximately 1 year later, the patients underwent the palatoplasty. At that time, the soft palatal adhesion was incised; with this exception, the palatoplasty was performed in accord with the standard procedure.

### Study design

**Fig 2:**
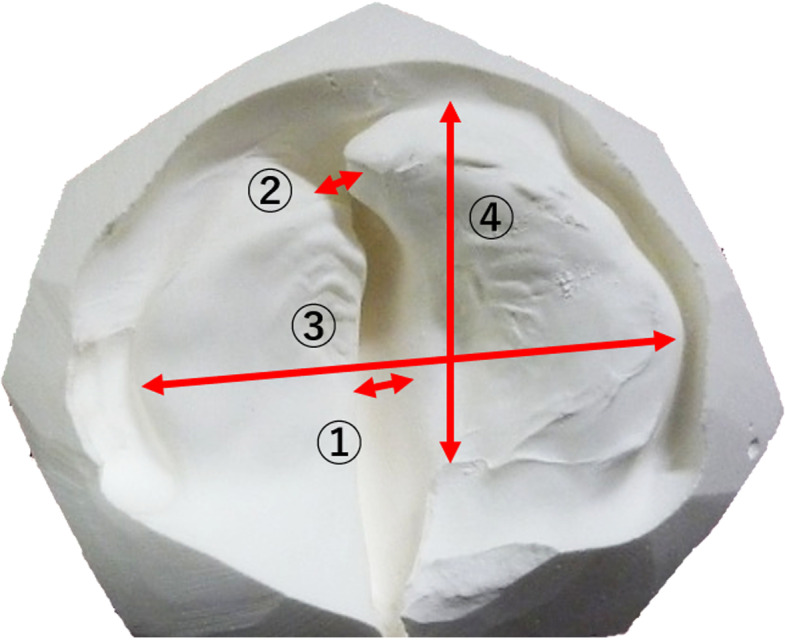
Measurement of the patients’ dental cast models. (1) Cleft width at the maxillary tuberosity. (2) Alveolar cleft width. (3) Maxillary dental arch width. (4) Maxillary dental arch length. The measurements were conducted at the patient’s birth, at the time of cheiloplasty, and at the time of palatoplasty.

Using the patients’ medical records and dental cast models, we conducted a comparative analysis between the patients for whom VA was used during their cleft lip repair (VA group) and those for whom VA was not used, as the control group (non-VA group). The following parameters were investigated: birth weight, cleft width at birth, alveolar cleft width, dental arch width of the maxilla, dental arch length of the maxilla, cleft width at lip repair, alveolar cleft width at lip repair, dental arch width of the maxilla at lip repair, dental arch length of the maxilla at lip repair, body weight at palatoplasty, age at palatoplasty, cleft width at palatoplasty, alveolar cleft width at palatoplasty, dental arch width of the maxilla at palatoplasty, dental arch length of the maxilla at palatoplasty, and the presence of OME at palatoplasty. The width measurements were taken using calipers on the dental cast models obtained at birth (initial visit), lip repair, and palatoplasty (Fig 2). The presence/absence of OME was evaluated at our hospital’s Department of Otorhinolaryngology the day before each patient’s palatoplasty.

### Statistical analyses

All of the statistical analyses were conducted with R ver. 4.3.2 (R Foundation for Statistical Computing, Vienna, Austria) and IBM SPSS Statistics ver. 24.0 (IBM, Armonk, NY) software. Pearson’s chi-square test and a two-tailed t-test were used. The level of significance was set at p < 0.05.

## Results

The cases of a total of 41 patients (27 males and 14 females) with the mean age 16.02 ± 2.28 months were analyzed. The VA group (n = 20) was fourteen males and six females, and the non-VA group (n = 21) was thirteen males and eight females. The age at palatoplasty was 16.65 ± 2.67 months in the VA group and 15.43 ± 1.62 months in the non-VA group (p = 0.01). The body weight at the time of palatoplasty was 9.73 ± 0.95 kg in the VA group and 9.26 ± 0.94 kg in the non-VA group (p = 0.12). Table 1 summarizes the groups’ demographic data.

**Table 1:** The number, age, and weight of the patients with cleft lip and palate in the velar adhesion VA group and non-VA group.

Variable	Non-VA (n = 21, 51.21%)	VA (n = 20, 48.78%)	Total (n = 41)	p-value
**Age at cheiloplasty, months**	4.43 (1.05)	4.80 (1.08)	4.61 (1.08)	>0.05
**Age at palatoplasty, months**	15.91 (2.71)	17.48 (3.74)	16.71 (3.37)	>0.05
**Duration between cheiloplasty and palatoplasty, months**	11.00 (1.54)	11.85 (2.26)	11.41 (1.98)	>0.05
**Body weight at palatoplasty, kg**	9.25 (0.94)	9.73 (0.95)	9.49 (0.98)	>0.05

The data are the number of patients (%) or mean (SD). P-values were calculated using a t-test.

**Fig 3:**
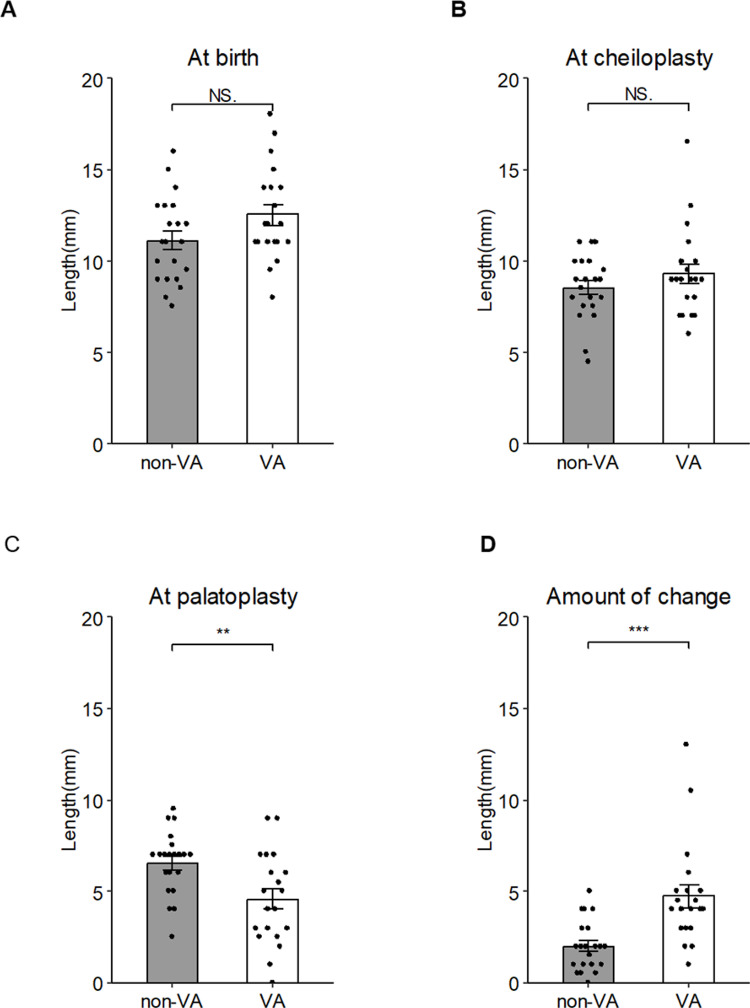
The cleft width at three timepoints: (A) at the patient’s birth, (B) at cheiloplasty, and (C) at palatoplasty. (D) The amount of change calculated from the data of the cleft width at the cheiloplasty and palatoplasty. Data are mean ± SD of each group. *p < 0.05, **p < 0.01 vs. non-VA.

The cleft width at birth was 12.53 ± 2.51 mm in the VA group and 11.13 ± 2.27 mm in the non-VA group, with no significant between-group difference (p = 0.07) (Fig 3A). Similarly, at the time of lip repair, the cleft width was 9.30 ± 2.37 mm in the VA group and 8.55 ± 1.73 mm in the non-VA group, a nonsignificant difference (Fig 3B). However, we identified a significant difference in the cleft width at the time of palatoplasty, with the cleft width in the VA group significantly narrower at 4.58 ± 2.44 mm compared to 6.55 ± 1.70 mm in the non-VA group (p = 0.01) (Fig 3C). Based on the calculation of the change in cleft width from cheiloplasty to palatoplasty, a reduction of 4.73 ± 2.73 mm was observed in the VA group compared to a reduction of 2.00 ± 1.35 mm in the non-VA group. This reduction was significantly greater in the VA group (p < 0.01) (Fig 3D).

**Fig 4:**
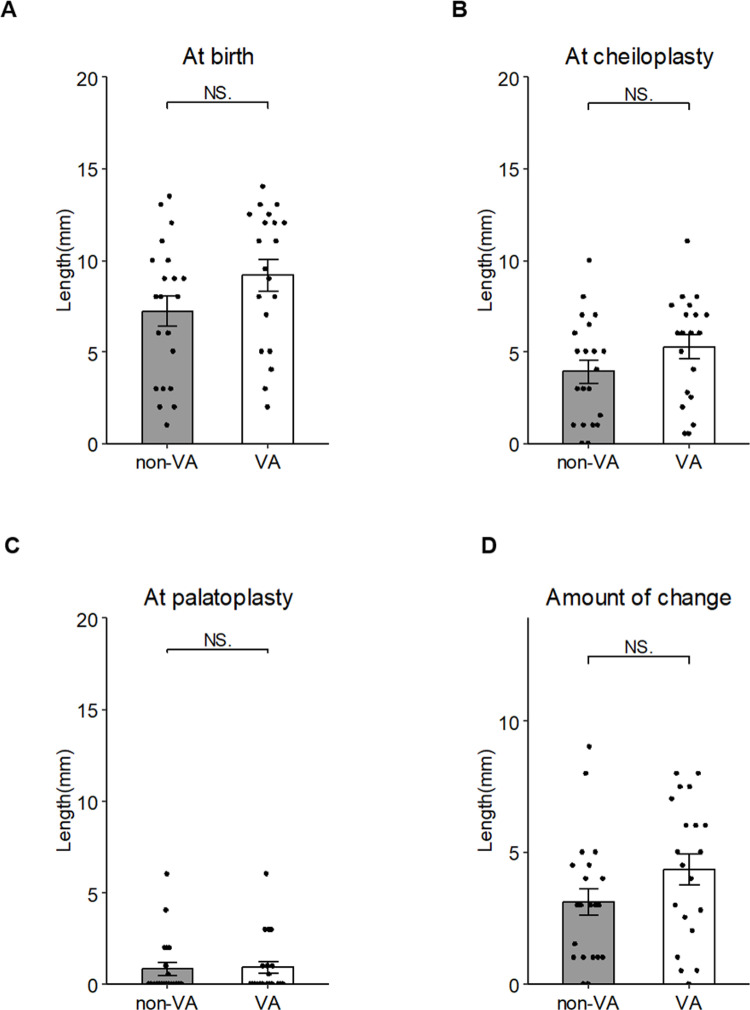
The alveolar width at three timepoints: (A) at the patient’s birth, (B) at cheiloplasty, and (C) at palatoplasty. (D) The amount of change calculated from the data of the alveolar width at the cheiloplasty and palatoplasty. Data are mean ± SD of each group. *p < 0.05, **p < 0.01 vs. non-VA.

Regarding the alveolar cleft width, no significant difference was observed between the groups at birth: 9.18 ± 3.63 mm in the VA group and 7.21 ± 3.73 mm in the non-VA group (Fig 4A). Similarly, no significant difference was found between the groups in alveolar cleft width at the time of cheiloplasty or palatoplasty: 5.27 ± 2.82 mm and 0.93 ± 1.57 mm in the VA group, and 3.95 ± 2.77 mm and 0.88 ± 1.55 mm in the non-VA group, respectively (Fig 4B and C). The changes in alveolar cleft width were also calculated, with reductions of 4.34 ± 2.60 mm in the VA group and 3.12 ± 2.34 mm in the non-VA group, a nonsignificant difference (Fig 4D).

**Fig 5:**
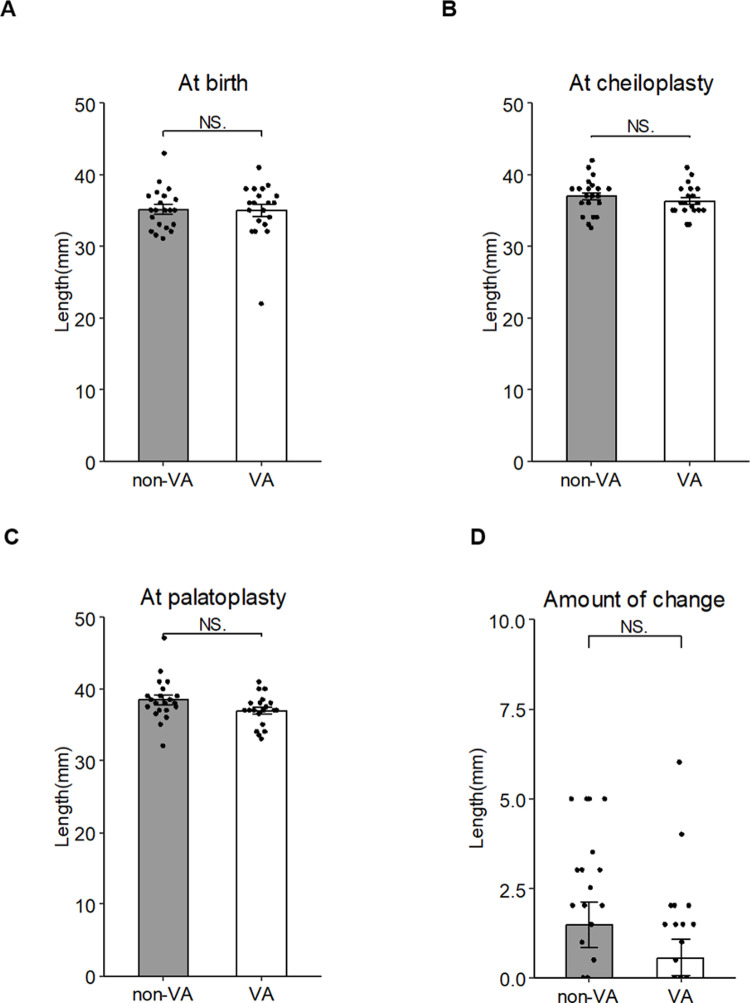
The dental arch width at three timepoints: (A) at the patient’s birth, (B) at cheiloplasty, and (C) at palatoplasty. (D) The amount of change calculated from the data of the dental arch width at the cheiloplasty and palatoplasty. Data are mean ± SD of each group. *p < 0.05, **p < 0.01 vs. non-VA.

To investigate the impact of VA on maxillary growth inhibition, we conducted measurements of the dental arch width and dental arch length. No significant between-group difference was observed for the dental arch width at birth: 35.00 ± 3.81 mm in the VA group and 35.14 ± 2.82 mm in the non-VA group (Fig 5A). The dental arch width at the time of cheiloplasty was 36.38 ± 2.07 mm in the VA group and 37.00 ± 2.46 mm in the non-VA group, a nonsignificant difference (Fig 5B). Similarly, no significant difference was observed in dental arch width at the time of palatoplasty: 36.95 ± 2.12 mm in the VA group and 38.48 ± 2.89 mm in the non-VA group (Fig 5C). The change in dental arch width from cheiloplasty to palatoplasty was 0.58 ± 2.23 mm in the VA group and 1.48 ± 2.81 mm in the non-VA group, another nonsignificant difference (Fig 5D).

**Fig 6:**
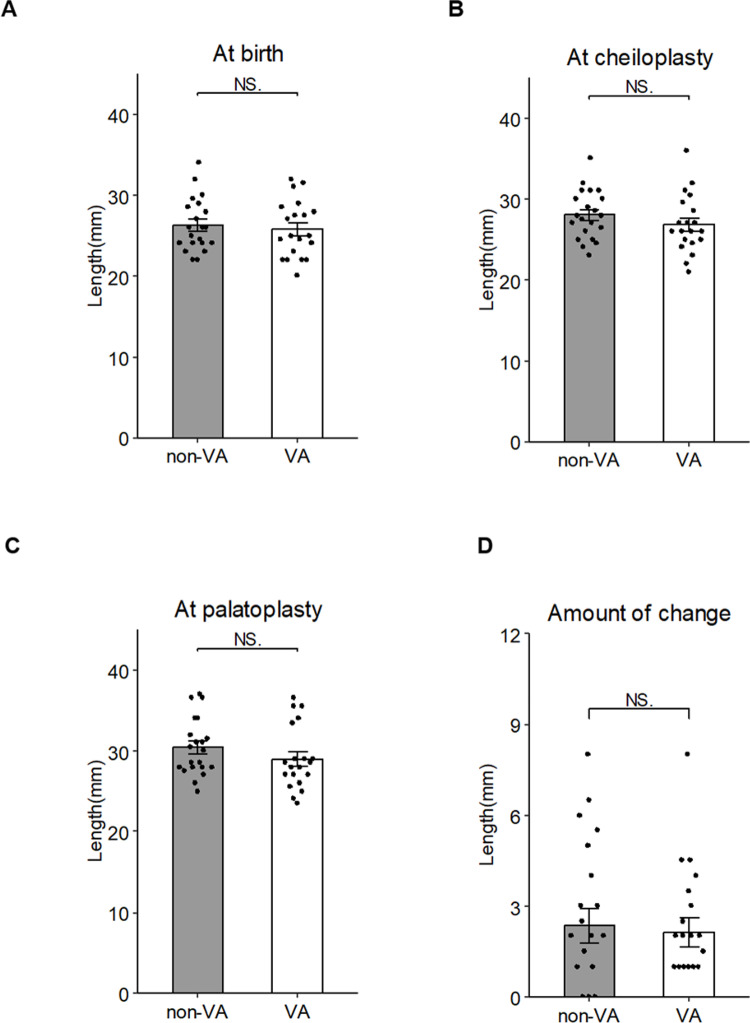
The dental arch length at three timepoints: (A) at the patient’s birth, (B) at cheiloplasty, and (C) at palatoplasty. (D) The amount of change calculated from the data of the dental arch length at the cheiloplasty and palatoplasty. Data are mean ± SD of each group. *p < 0.05, **p < 0.01 vs. non-VA.

Concerning the dental arch length of the maxilla, no significant between-group difference was detected: 25.80 ± 3.50 mm and 26.26 ± 3.21 mm in the VA and non-VA groups, respectively (Fig 6A). Similarly, no significant between-group difference was shown between the groups at the time of cheiloplasty: 26.85 ± 3.51 mm in the VA group and 28.05 ± 2.94 mm in the non-VA group (Fig 6B). At the time of palatoplasty, the dental arch length was 29.00 ± 3.83 mm in the VA group and 30.40 ± 3.43 mm in the non-VA group, a nonsignificant difference (Fig 6C). The change in dental arch length from cheiloplasty to palatoplasty was 2.15 ± 2.05 mm in the VA group and 2.36 ± 2.60 mm in the non-VA group, with no significant difference between the groups (Fig 6D).

Our investigation of the relationship between OME and VA revealed the OME occurred in 12 of the patients in the VA group (60.0%), and not in the other eight patients in this group. Notably, in the non-VA group, OME was observed in 19 cases (90.48%), with only two cases (9.52%) not presenting with OME. The prevalence of OME in the VA group was significantly lower than that in the non-VA group (p = 0.023) (Table 2). Our examination of the presence of tympanostomy tube insertion revealed that 10 of the 20 patients (50%) in the VA group underwent tympanostomy tube insertion, whereas 18 of the 21 patients (85.71%) in the non-VA group underwent the insertion of a tympanostomy tube (Table 2). The rate of tympanostomy tube insertion was significantly lower in the VA group (p = 0.014).

**Table 2:** (A) The incidence of OME in the VA group compared to the non-VA group. (B) The rate of tympanostomy tube insertion in the VA group and non-VA group.

		(A) OME			p-value
		Yes	No	Total	
		No. (%)	No. (%)	No. (%)	
**VA**	Yes	12 (60.00)	8 (40.00)	20 (100)	0.023*
	No	19 (90.91)	2 (9.09)	21 (100)	
		**(B) Tubing**			**p-value**
**VA**	Yes	10 (50.00)	10 (50.00)	20 (100)	0.014*
	No	18 (85.71)	3 (14.29)	21 (100)	

* Results of Pearson’s chi-square test

VA: velar adhesion, OME: otitis media with effusion. Pearson’s chi-square test was performed.

## Discussion

Children with CLP may face significant functional challenges due to the presence of the palatal cleft, including potential language impairments and susceptibility to OME. For these patients, a palatoplasty is a crucial procedure aimed at addressing such problems. Performing a precise palatoplasty is of paramount importance for surgeons, given the significant impact it has on patients’ lives. Over the years, numerous clinicians have reported various modifications and techniques regarding palatoplasty [10–15]. We focus herein on the surgical technique known as VA, which involves suturing only the mucosal surface of the soft palate during cleft lip repair in patients with CLP, with the aim of facilitating a future palatoplasty. Despite its potential utility, there have been few reports about VA, and its specific efficacy and detailed effects have been unclear.

The VA procedure is simple and can be completed in approx. 10 minutes, with minimal burden on the patient. It is concluded before the commencement of lip repair, minimizing patient discomfort as it is performed under general anesthesia. First, local anesthesia with a small amount of 2% lidocaine with 1:200,000 epinephrine is administered, followed by incisions made to the level of the muscle layer along the edge of the cleft palate. The incision extends from the base of the uvula to the maxillary tuberosity. The mucosa is then sutured with absorbable threads, with a few stitches on the oral and nasal sides, respectively. This technique relies on the tension created by suturing the mucosa together, and thus no muscle repair is performed; in addition, only the mucosal surface is sutured.

However, during a palatoplasty, attention must be paid to scar tissue manipulation, as the adhesion site must be detached and re-sutured during palate formation, resulting in scar tissue. Specifically, during the incision and dissection of the cleft palate edge, caution is required to identify the scar tissue layer, which differs from the preoperative mucosa. Extra care must be taken during suturing to avoid creating a fistula, as scar tissue is being sutured together. Our department has extensive experience with VA procedures, and we have not encountered any cases of fistula formation following palatoplasty in patients for whom VA is applied.

One of the crucial functional issues for patients with CLP is language impairment [17]. Although a palatoplasty aims to improve language function, postoperative fistula formation or insufficient extension of the soft palate may hinder the acquisition of normal speech function. The success of a palatoplasty is crucial from a linguistic standpoint. VA is a highly valuable method that contributes to the success of a palatoplasty. Oyama et al. reported that the width of the cleft palate at the time of palatoplasty was smaller in patients who underwent VA [16]. While their research was limited by a small sample size, the groups in our present patient population were each ≥20 cases. In addition, we calculated the amount of change and demonstrated the effect of VA on the cleft width.

Our analyses revealed a 49.19% decrease in the cleft width during the palatoplasties in the VA group, showing a significant difference compared to the non-VA group. It has become evident that there is a significant reduction in cleft width at the junction of the hard and soft palates. Although we also observed a decrease in the alveolar cleft width of the VA patients, similar results were found in the non-VA group, suggesting that the effect may be primarily due to lip pressure after lip repair [18]. Simultaneous cheiloplasty and VA surgery has a combined effect: the cheiloplasty addresses the anterior aspect, and the VA affects the posterior aspect, resulting in an overall reduction in cleft width during the palatoplasty and facilitating the procedure.

In a conventional palatoplasty, the formation of palatal scars may lead to maxillary hypoplasia [19]. The same concern applies to VA, which also results in scar formation on the palate. To investigate whether the application of VA affects maxillary growth, we compared the growth of the maxilla from the cheiloplasty with VA to the palatoplasty between the VA and non-VA groups, and our analysis did not reveal any significant difference in maxillary growth between the two groups. Since VA involves superficial scarring limited to the mucosal surface, the size of the scar is minimal, suggesting minimal impact on palatal growth. Moreover, as the procedure affects primarily soft tissues such as the soft palate, it minimizes intrusion into the palatal bone, thereby mitigating the risk of inhibiting growth due to scar formation.

The anatomical structure of clefting in the palate in individuals with CLP is known to predispose them to OME complications [6], which is one of the most frequent complications in patients with CLP. A comorbidity rate of OME exceeding 90% has been described [7]. OME has been reported to be associated primarily with dysfunctional soft palate muscles, which in turn affects the patency of the eustachian tube [20]. In individuals with a cleft palate, the eustachian tube orifice is often directly exposed to the oral cavity, potentially leading to a contaminated environment that is conducive to the development of OME. VA surgery, performed around 3–6 months of age during lip repair, results in closure of the soft palate, providing coverage to the eustachian tube orifice compared to cases without VA. A reduced incidence of OME following palatoplasty has been observed in several studies [21–23], suggesting that a similar effect could be expected with VA. Furthermore, unlike the conventional palatoplasty, VA performed simultaneously with lip repair allows for the early modification of the anatomical structure. Performing VA does not involve muscle repair, and the adhesion of the mucosa of the soft palate may alter the direction of muscles to some extent, potentially contributing to changes in the tissue environment around the eustachian tube orifice.

Since OME can affect an individual’s hearing and pronunciation, reducing the incidence of OME by conducting VA could be beneficial for patients with CLP. A hearing impairment can have significant impacts on infant patients, including delayed language development and reduced learning abilities [24]. Our present findings indicate that VA is a highly useful surgical approach for preventing OME, and further research is needed to explore the relationship between VA and OME.

This study has several limitations. First, the sample size was relatively small, with only 41 patients meeting the selection criteria. While the study suggests that VA contributes to cleft width reduction and a lower incidence of OME, the analysis was based on cases from a single institution, limiting the generalizability of the findings. Larger cohort studies will be necessary to confirm these results. Second, although significant differences were observed in cleft width reduction and the incidence of OME, this study did not include long-term follow-up data on maxillary growth or other functional aspects, such as speech outcomes. A longitudinal study is required to evaluate the long-term impact of VA on maxillary development and patient prognosis.

## Conclusions

Velar adhesion is a relatively straightforward surgical procedure that imposes a minimal burden on patients. The results of our present analyses suggest that VA significantly reduces the cleft width at the junction of the hard and soft palates. VA also presents the potential for mitigating complications such as OME, thus reducing the incidence rate of OME.
